# A Risk Score to Predict Type 2 Diabetes Mellitus in an Elderly Spanish Mediterranean Population at High Cardiovascular Risk

**DOI:** 10.1371/journal.pone.0033437

**Published:** 2012-03-19

**Authors:** Marta Guasch-Ferré, Mònica Bulló, Bernardo Costa, Miguel Ángel Martínez-Gonzalez, Núria Ibarrola-Jurado, Ramon Estruch, Francisco Barrio, Jordi Salas-Salvadó, for the PREDI-PLAN Investigators

**Affiliations:** 1 Human Nutrition Unit, Faculty of Medicine and Health Sciences, IISPV, Universitat Rovira i Virgili, Reus, Spain; 2 CIBERobn Physiopathology of Obesity and Nutrition, Institute of Health Carlos III, Madrid, Spain; 3 Department of Preventive Medicine and Public Health, University of Navarra, Pamplona, Spain; 4 Catalan Health Institute, Jordi Gol Primary Care Research Institute, Catalunya, Spain; 5 Department of Internal Medicine, Hospital Clinic, University of Barcelona, Barcelona, Spain; Fundación para la Prevención y el Control de las Enfermedades Crónicas No Transmisibles en América Latina (FunPRECAL), Argentina

## Abstract

**Introduction:**

To develop and test a diabetes risk score to predict incident diabetes in an elderly Spanish Mediterranean population at high cardiovascular risk.

**Materials and Methods:**

A diabetes risk score was derived from a subset of 1381 nondiabetic individuals from three centres of the PREDIMED study (derivation sample). Multivariate Cox regression model ß-coefficients were used to weigh each risk factor. PREDIMED-personal Score included body-mass-index, smoking status, family history of type 2 diabetes, alcohol consumption and hypertension as categorical variables; PREDIMED-clinical Score included also high blood glucose. We tested the predictive capability of these scores in the DE-PLAN-CAT cohort (validation sample). The discrimination of Finnish Diabetes Risk Score (FINDRISC), German Diabetes Risk Score (GDRS) and our scores was assessed with the area under curve (AUC).

**Results:**

The PREDIMED-clinical Score varied from 0 to 14 points. In the subset of the PREDIMED study, 155 individuals developed diabetes during the 4.75-years follow-up. The PREDIMED-clinical score at a cutoff of ≥6 had sensitivity of 72.2%, and specificity of 72.5%, whereas AUC was 0.78. The AUC of the PREDIMED-clinical Score was 0.66 in the validation sample (sensitivity = 85.4%; specificity = 26.6%), and was significantly higher than the FINDRISC and the GDRS in both the derivation and validation samples.

**Discussion:**

We identified classical risk factors for diabetes and developed the PREDIMED-clinical Score to determine those individuals at high risk of developing diabetes in elderly individuals at high cardiovascular risk. The predictive capability of the PREDIMED-clinical Score was significantly higher than the FINDRISC and GDRS, and also used fewer items in the questionnaire.

## Introduction

Type 2 diabetes mellitus (T2DM) is one of the most common chronic diseases worldwide, affecting more than 285 million people in 2010. Its prevalence has increased overwhelmingly in recent years in parallel to the obesity epidemics [1], [2]. Recently, it has been estimated that the number of adults with diabetes will increase between 2010 and 2030 by 69% and 20% in developing and industrialized countries, respectively [2]. The increase in the prevalence of T2DM across the world has become an important public health problem given that it ranks among the leading causes of blindness, renal failure lower limb amputation, and is also a significant risk factor for coronary heart disease and stroke, thus increasing mortality [3] and increasing human and financial public health costs [4].

Strong evidence from several studies has demonstrated that T2DM could be prevented by diet and other lifestyle modifications in high-risk individuals (reviewed by [5]) [6], [7]. Thus, it is important the earlier detection of individuals at high risk for diabetes to lead to this target population the intensive preventive interventions [8], [9].

Simple prediction tools that can identify those individuals at high risk of developing T2DM could reduce the cost and inconvenience of screening [10]. Several multivariate risk scores based on anthropometric measurements, lifestyle factors and simple laboratory markers have been developed to identify healthy individuals at high risk [8], [9], [11], [12], but the great majority of these scores have been designed and validated using either North American or European study populations [13]. Furthermore, most studies have been done in healthy young or middle-aged individuals from the general population. Because various diabetes risk factors (e.g. hypertension, abdominal obesity, beta-cell function decline…) could change with age and age has been considered an important non modifiable risk factor of incident diabetes [8], [11]. It is important to derive a risk score for elderly population, who is usually attended by Primary Care clinicians.

To our knowledge, no risk score for predicting incident diabetes have been developed in elderly Spanish individuals. Therefore, the main aim of the present study was to develop a diabetes risk score for elderly Spanish population at high cardiovascular risk using information routinely collected or simple laboratory measures, which could effectively predict incident diabetes and be applied in daily clinical practice. The score was developed in a cohort of non-diabetic individuals from the PREDIMED study and tested in an independent Spanish cohort (DE-PLAN-CAT project). We also assessed and compared the predictive capability of PREDIMED diabetes risk scores to other diabetes risk scores developed in European populations in both cohorts.

## Materials and Methods

For PREDIMED Study the respective local institutional review boards (Hospital Universitari Sant Joan de Reus and Fundació Gol i Gurina) approved the study protocol and all participants provided written informed consent. For DE-PLAN-CAT Project the research ethics board of the Jordi Gol Research Institute (Barcelona, Spain) approved the protocol, and all participants gave written informed consent.

The PREDIMED study (PREvención con DIeta MEDiterránea) is a large, parallel-group, multicenter, randomized, controlled clinical trial which aims to assess the effects of the Mediterranean diet on the primary prevention of cardiovascular disease (CVD) (www.predimed.org and www.predimed.es). The PREDIMED study is being conducted in Spain. Recruitment took place between October 2003 and January 2009, and the 7447 participants were randomly assigned to one of three interventions (two Mediterranean diets enriched with extra virgin olive oil or mixed nuts and a control low-fat diet designed by the American Heart Association). The design and methods used in the PREDIMED study have been described elsewhere [14].

The present report describes a nested study aimed at longitudinally assess the predictive value of classical markers of incident diabetes and develop a diabetes risk score for elderly Spanish individuals. The 1381 candidates included in the derivation sample were all non diabetic Caucasian individuals recruited in PREDIMED centres in Navarra (n = 650), Reus (n = 418) and Barcelona (n = 313). They were men aged 55-80 years and women aged 60-80 years, who were free of CVD at baseline but fulfilled at least three or more coronary heart disease risk factors: current smoking, hypertension (blood pressure >140/90 mmHg or treatment with antihypertensive medication), hypertriglyceridemia (serum triglycerides ≥150 mg/dL or requiring treatment), low plasma HDL-cholesterol (≤40 mg/dL in men and ≤50 mg/dL in women), overweight or obesity (BMI≥25 kg/m^2^), and family history of premature CVD (≤55 years in men and ≤60 years in women). The exclusion criteria for the PREDIMED study were any severe chronic illness, previous history of CVD, alcohol or drug abuse, BMI≥40 kg/m^2^ and history of allergy or intolerance to olive oil or nuts. The participants in the derivation sample were free of diabetes at baseline because one purpose was to assess new onset T2DM during follow-up. The median follow-up was 6.0 years (mean 4.75 years, minimum 3 months - maximum 8.2 years). At baseline examination and yearly in follow-up visits, trained personnel performed anthropometric and blood pressure measurements and obtained samples of fasting blood. Weight and height were measured with light clothing and no shoes with calibrated scales and a wall-mounted stadiometer, respectively; waist circumference was measured midway between the lowest rib and the iliac crest using an anthropometric tape; blood pressure was measured using a validated oscillometer [Omron HEM705CP, Hoofddorp, Netherlands] in triplicate with a 5-min interval between each measurement, and the mean of these values was recorded. We also administered a 137-item validated food frequency questionnaire [15]; the validated Spanish version of the Minnesota Leisure Time Physical Activity Questionnaire [16]; and a 47-item questionnaire about education, lifestyle, history of illnesses and medication use. Samples of serum, EDTA plasma, and urine were coded, shipped to central laboratories, and stored at −80°C until analysis. Centralized laboratory analyses were performed on frozen serum samples obtained in fasting conditions. Serum glucose, cholesterol, and triglyceride levels were measured using standard enzymatic automated methods. HDL-cholesterol was measured by enzymatic procedure after precipitation.

The validation sample was an active public health program (DE-PLAN) carried out in Catalonia (Spain) [17]. The design of DE-PLAN-CAT/PREDICE has been described elsewhere [18], [19]. This cohort was used to externally validate the predictive capability of the scores previously developed in the derivation sample. The derivation sample included all the 552 participants in the DE-PLAN-CAT project. They were also Spanish Caucasian individuals without diabetes, but younger (45–75 years) than the individuals in the PREDIMED population. They were screened using the Finnish diabetes risk score – FINDRISC questionnaire and a 2-h oral glucose tolerance test, and the subjects were characterized according to their future risk of T2DM [8]. All subjects who had a high risk of T2DM (FINDRISC score >14 and/or prediabetes diagnosis criteria at blood test) were randomized to two lifestyle interventions and included in the present analysis. All subjects with prevalent diabetes, severe psychiatric disease, chronic kidney or liver disease or blood disorders were excluded. The DE-PLAN-CAT intervention consisted of two steps (initial and further reinforcement) and two elective interventions (informative or intensive). The usual care intervention consisted of giving information to the participants about diet and cardiovascular health but without an individual program; on the other hand, the intensive DE-PLAN-CAT educational program consisted of a six-hour educational program recommending the same Mediterranean diet used in the PREDIMED study. Participants were followed for a median period of 4.2 years of follow-up that was almost the same as in the PREDIMED study (mean 3.8 years, minimum 4 months - maximum 5.3 years) [18], [19].

The information gathered was comparable to the PREDIMED study; all participants underwent a physical examination and medical history, and gave information about their smoking status, diet, alcohol consumption habits and physical activity. Lipid profile and glucose determinations were also performed at baseline.

Methods and parameters used for the diagnosis of incident diabetes were the same in both studies. New onset diabetes was diagnosed using American Diabetes Association criteria [20], namely fasting plasma glucose (FPG) ≥126 mg/dL (≥7 mmol/L) or 2-h plasma glucose ≥200 mg/dL (≥11.1 mmol/L) after a 75 g oral glucose load, measured yearly. A second test using the same criteria was required for confirmation. Cases were ascertained by a clinical Event Committee in the PREDIMED study.

We developed a series of multivariate Cox regression models to produce risk functions for detecting incident diabetes in the PREDIMED Study. We developed two different predictive models: one of them contained easily obtained clinical variables, such as anthropometric parameters (sex, age, BMI, waist circumference), lifestyle factors (smoking status, alcohol consumption, physical activity) and categorical nutritional variables (consumption of vegetables, fruit, red meat, fish, coffee and other foods); the other contained the same variables but also components of the Metabolic Syndrome, defined by the updated criteria of the National Cholesterol Education Program Adult Treatment Panel III (NCEP, 2001). Abdominal obesity was defined as a waist circumference of ≥102 cm in men or ≥88 cm in women. Low HDL-cholesterol was defined as <40 mg/dL (<0.9 mmol/L) in men or <50 mg/dL (<1.2 mmol/L) in women. The MS (Metabolic Syndrome) was considered to have a component of hypertriglyceridemia when triglyceride concentrations were ≥150 mg/dL (≥1.7 mmol/L) or when subjects were receiving fibrate treatment. Hypertension was defined as a blood pressure level of ≥130/85 mmHg or when subjects were receiving antihypertensive medication. Hyperglycemia was defined as a FPG concentration of ≥100 mg/dL (≥5.4 mmol/L). Finally, to develop the diabetes risk score we selected only those variables that were statistically significant (P<0.05). The risk factors considered in the first model (the PREDIMED-personal model) were: categories of BMI (≥27 or <27 kg/m^2^), smoking status (current smoker or non-smoker), alcohol consumption (≥3 standard drink units for men or ≥1.5 for women or less than these amounts), a family history of T2DM (only when it was present in a first degree relative, including mother, father or siblings) and the presence or absence of hypertension at baseline. The second model (the PREDIMED-clinical model) included the same variables as in the first model but also a categorical variable of FPG concentrations. Coefficients (β) of the models were used to assign a weight value for each variable. The PREDIMED-personal and the PREDIMED-clinical diabetes risk scores were calculated as the sum of these weights. The sensitivity (that is to say, the probability that the test will be positive for subjects who will develop diabetes in the future) and, specificity (the probability that the test will be negative for subjects without diabetes) with 95% CIs were calculated for the scores. Then, receiver-operating characteristic (ROC) curves were plotted for the scores; the sensitivity was plotted on the y-axis, and the false positive rate (1-specificity) was plotted on the x-axis. If the AUC is 1.0, there is a cut point for the variable at which there is perfect discrimination into cases and no cases of incident diabetes. The scores derived in the PREDIMED study subset were tested in the DE-PLAN-CAT project as an external validation sample to assess their diagnostic properties on an independent sample; for this purpose we used areas under the curve (AUC). We first estimated each subject's probability of developing diabetes on the basis of the derived risk functions produced in the PREDIMED study. We also compared the performance of our scores with other scores by assigning all our subjects a FINDRISC score and a GDRS. The questions that we used to determine the GDRS were the same as in the original score.

The German Diabetes risk score included anthropometric parameters and lifestyle factors such as categories of age, waist circumference and height; being or not physically active, have been diagnosed of high blood pressure, the intake of dietetics fiber, the consumption of meat, the intake of alcohol and coffee, and the smoking habit [11]. The FINDRISC Score included also anthropometric parameters and lifestyle factors (categories of age, BMI, and waist circumference; being or not physically active, the frequency of eating fruit, vegetables and berries; have ever taken antihypertensive medication regularly, having had a history of high blood glucose and family history of diabetes) [8]. However, we were obliged to assign an adapted FINDRISC score, because one of the variables was not collected in our samples. We calculated the FINDRISC personal score without using the question on the personal history of high blood glucose, and in the FINDRISC clinical score we included the FPG concentrations instead of the information about the personal history of high blood glucose. We repeated these analyses in the PREDIMED and DE-PLAN-CAT cohorts.

The level of significance for all statistical tests was P<0.05 for bilateral contrasts. Data were analyzed using the SPSS statistical package version 17.0. To assess statistical differences between ROC curves, EPIDATA 3.1 software was used.

## Results

The baseline characteristics and differences between both projects and those individuals in each project who developed incident diabetes and those who did not are summarized in Table 1. A total of 41.4% of the 1381 non diabetic subjects in this subset from three PREDIMED centres were men, 23.1% had a family history of diabetes and 17.6% were current smokers. The mean age of the population was 67 years. Of all the subjects analyzed, 155 (11.2%) developed diabetes during the mean of 4.75-year follow-up. A total of 35.5% of the subjects who developed new onset T2DM reported a family history of diabetes and 26.5% were current smokers; FPG was higher in this group than in their non-incident diabetic counterparts (P<0.001) (Table 1). In the DE-PLAN-CAT external validation sample, 33.3% of individuals were men, 69.2% had a family history of diabetes and 37.5% were current smokers; the mean age was similar (62 years) to the PREDIMED sample. Of the 552 non-diabetic participants, 124 (22.4%) developed diabetes during the mean of 4.2-year follow-up. Of the individuals with incident diabetes, 62.1% reported a family history of diabetes, and 43.5% were current smokers; FPG was also higher than among their non-diabetic counterparts (p<0.001).

**Table 1:** Baseline characteristics of the study participants.

	PREDIMED derivation sample (3 centres)				DE-PLAN-CAT validation sample				
Characteristic	Total subjects (n = 1381)	NIDM (n = 1226)	IDM (n = 155)	Pvalue^a^	Total subjects (n = 552)	NIDM (n = 428)	IDM (n = 124)	Pvalue^a^	Pvalue^b^
Age, years	67 (6)	67 (6)	66 (5)	0.142	62 (7)	62 (8)	62 (7)	0.653	<0.001
Men, n (%)	572 (41.4)	499 (40.7)	73 (47.1)	0.128	184 (33.3)	131 (30.6)	53 (42.7)	0.012	0.001
Weight, kg	75.3 (11.0)	75.0 (11.0)	78.3 (11.2)	<0.001	78.9 (12.9)	78.0 (12.4)	81.9 (14.1)	0.003	<0.001
BMI, kg/m^2^	29.4 (3.2)	29.3 (3.2)	30.1 (3.1)	0.004	31.2 (4.6)	30.9 (4.4)	32.0 (5.1)	0.027	<0.001
Waist circumference, cm	97.9 (9.9)	97.5 (9.8)	101.6 (9.4)	<0.001	100.6 (10.6)	99.9 (10.2)	102.7 (11.7)	0.012	<0.001
Family history of diabetes, n (%)	319 (23.1)	264 (21.5)	55 (35.5)	<0.001	382 (69.2)	305 (71.3)	77 (62.1)	0.052	<0.001
Current smoker, n (%)	243 (17.6)	202 (16.5)	41 (26.5)	0.002	207 (37.5)	153 (35.7)	54 (43.5)	0.114	<0.001
Systolic blood pressure, mmHg	151.4 (20.2)	151.1 (20.2)	154.1 (19.6)	0.079	134.0 (14.3)	133.5 (13.6)	135.5 (16.4)	0.209	<0.001
Diastolic blood pressure, mmHg	85.6 (10.2)	85.4 (10.2)	87.0 (10.3)	0.078	80.1 (9.1)	79.5 (9.1)	82.0 (9.2)	0.009	<0.001
Total cholesterol, mg/dL	223.5 (37.1)	224.0 (36.4)	219.3 (41.8)	0.138	211.2 (36.2)	211.6 (36.2)	210.1 (36.6)	0.684	0.084
HDL cholesterol, mg/dL	56.8 (14.2)	57.3 (14.3)	53.2 (12.5)	<0.001	58.2 (14.9)	59.1 (15.5)	55.1 (12.2)	0.003	0.054
LDL cholesterol, mg/dL	142.1 (32.2)	142.7 (32.3)	137.6 (31.1)	0.072	127.9 (32.1)	127.8 (31.7)	128.2 (33.5)	0.901	<0.001
Triglycerides, mg/dL	133.4 (72.0)	130.7 (64.6)	155.3 (112.9)	0.009	126.9 (68.2)	122.2 (59.9)	143.4 (89.5)	0.016	0.069
Fasting glucose, mg/dL	96.6 (15.0)	94.6 (13.4)	112.3 (17.7)	<0.001	94.1 (12.7)	91.9 (11.7)	101.8 (12.8)	<0.001	0.001

*Abbreviations: BMI, body mass index. HDL, high-density lipoprotein; IDM, incident Diabetes Mellitus; LDL, low-density lipoprotein, NIDM, non incident Diabetes Mellitus.*

*Data expressed as mean (SD) or number (percent). P value^a^ of the difference between incident diabetic and non-incident diabetic subjects. P value^b^ of the difference between individuals in the derivation sample and individuals in the validation sample (T-test for continuous variables or Chi-square tests for categorical variables).*

Relative risks (95% CI), ß-coefficients and points allocated to each variable derived from Cox regression models were shown in Table 2. Significant predictors of T2DM in models were selected to develop the diabetes risk scores. Statistically significant predictors of incident T2DM in the PREDIMED-personal model were BMI≥27 kg/m^2^ [HR (CI): 1.62 (1.07 to 2.42)], current smoking [HR (CI): 1.54 (1.06 to 2.24)], family history of T2DM [HR (CI): 2.02 (1.45 to 2.81)], and alcohol consumption [HR (CI): 1.94 (1.32 to 2.85)]. The PREDIMED-personal model included these variables and also hypertension [HR (CI): 2.06 (0.96 to 4.45)], although it did not add any further predictive power to the statistical model. Even so, we included it in our scores since it is considered to be an important predictor of incident diabetes and it was borderline significant in the personal model and significant (P = 0.033) in the clinical model. The PREDIMED-clinical model included the same variables as the personal model, but also FPG [HR (CI): 6.88 (4.76 to 9.94)], which was the strongest predictor of incident diabetes. The value for each variable of the PREDIMED scores was defined from the ß-coefficient of the personal model, except for the FPG (Table 2). For ß = 0.01–0.20, the weight was 1; for ß = 0.21–0.8, the weight was 2; for ß = 0.81–1.20, the weight was 3; for ß = 1.21–2.20, the weight was 4, and for ß>2.21 the weight was 5. The lowest category of each variable was given a weight of 0 whereas for the highest category, the score allocated to each variable was given. The total PREDIMED-personal score was calculated as the sum of risk factors and varied from 0 to 10 points. The PREDIMED-clinical score was the sum of all variables included in the clinical model and varied from 0 to 14 points.

**Table 2:** Baseline risk factors for incidence of type 2 diabetes in the PREDIMED cohort (Cox regression model).

	PREDIMED-personal model: n = 1381 (Incident diabetes: n = 155)				PREDIMED-clinical model: n = 1381 (Incident diabetes: n = 155)			
Risk factor	Hazard ratio (95% CI)	β	P value	Score	Hazard ratio (95% CI)	β	P value	Score
BMI≥27 kg/m^2^	1.62 (1.07–2.42)	0.486	0.022	2	1.37 (0.90–2.08)	0.315	0.140	2
Current smoker	1.54 (1.06–2.24)	0.435	0.023	2	1.72 (1.18–2.52)	0.547	0.005	2
Family history of T2DM	2.02 (1.45–2.81)	0.705	<0.001	2	1.65 (1.19–2.31)	0.506	0.003	2
Alcohol consumption	1.94 (1.32–2.85)	0.666	0.001	2	1.53 (1.04–2.26)	0.427	0.031	2
Hypertension	2.06 (0.96–4.45)	0.727	0.063	2	2.31 (1.06–5.00)	0.838	0.033	2
FPG≥100 mg/dL	-	-	-	-	6.88 (4.76–9.94)	1.929	<0.001	4
**TOTAL SCORE**	-	-	-	**10**	-	-	-	**14**

*Abbreviations: T2DM, Type 2 Diabetes Mellitus; BMI, body mass index; SDU, standard drink unit; CI, confidence interval; FPG, Fasting Plasma Glucose. One standard drink unit corresponds to 10 grams of pure alcohol. Alcohol consumption is considered when* ≥1.5 SDU in women or ≥3 in men. *Hypertension is considered when subjects use blood pressure medication or when pressure level ≥130/85 mmHg. All models were adjusted by intervention group. The score values were estimated on the basis of the β coefficients of the Cox regression models.*

In Table 3, the incidence of diabetes during follow-up was classified according to the score range in which individuals were categorized. It was high in the two upper categories of our clinical score in both the PREDIMED derivation sample and DE-PLAN-CAT validation sample. In the PREDIMED study, the percentage of individuals diagnosed with incident diabetes in the middle (5 to 9 points) and the highest (10 to 14 points) categories of the clinical score was 13.6% and 36.5% respectively. The P for trend of the score performance in the derivation sample was <0.001. A cut point of ≥6 in our clinical score identified 88.4% of the incident cases of diabetes. In the external validation sample (DE-PLAN-CAT cohort), the observed incidence was also high in the middle and the highest categories of our clinical score (P for trend <0.001). A total of 86% of subjects who developed new onset diabetes had 6 or more points based on our clinical score. The areas under the ROC curves of several diabetes risk scores assessed in both samples (derivation and validation) are shown in Table 4. Our clinical score reasonably predicted incident diabetes in the derivation sample (PREDIMED study subset) and had an AUC = 0.78. This AUC significantly outperformed the areas under the curve of our personal score (0.64), the FINDRISC personal and clinical scores (AUC = 0.58 and 0.71, respectively) and the GDRS (AUC = 0.59) when they were applied to the PREDIMED derivation sample (P<0.05 when the clinical score was compared to the other scores tested). Our personal score also had a higher AUC than the FINDRISC personal score and the GDRS (P<0.05). The sensitivity and specificity of our clinical score for identifying undiagnosed diabetes at a cutoff of ≥6 points were 72.2% and 72.5%, respectively. The positive predictive value was 25% and the negative predictive value was 95.4%.

**Table 3:** Diabetes incidence by categories of the Clinical Score during follow-up in the PREDIMED derivation sample and the DE-PLAN-CAT validation sample.

	PREDIMED derivation sample			DE-PLAN-CAT validation sample		
PREDIMED-clinical Score	Total subjects	Incident diabetes	%^a^	Total subjects	Incident diabetes	%^a^
0–4	658	18	2.7	132	18	13.6
5–9	556	76	13.6	303	58	19.1
10–14	167	61	36.5	117	48	41.0
P for trend	<0.001			<0.001		
**Total subjects**	1381	155		552	124	

a Percentage of individuals with incident diabetes in each category of PREDIMED-clinical Score.

**Table 4:** The predictive performance of different risk scores applied to the PREDIMED and the DE-PLAN-CAT cohorts.

	PREDIMED derivation sample (n = 1381)					DE-PLAN-CAT validation sample (n = 552)				
Diabetes risk score	AUC	Sensitivity (%) (95% CI)	Specificit (%) (95% CI)	PPV (%) (95% CI)	NPV (%) (95% CI)	AUC	Sensitivity (%) (95% CI)	Specificity (%) (95% CI)	PPV (%) (95% CI)	NPV (%) (95% CI)
PREDIMED-personal score	0.641†	52.9 (44.7–61.0)	70.4 (67.8–73.0)	18.5 (15.0–22.4)	92.2 (90.3–93.8)	0.507†	64.5 (55.4–72.9)	35.9 (31.4–40.7)	22.6 (18.3–27.3)	77.8 (71.3–83.4)
FINDRISC personal score	0.582* †	34.8 (27.4–42.9)	78.1 (75.7–80.4)	16.8 (12.9–21.3)	90.5 (88.5–92.2)	0.520†	61.2 (52.1–69.9)	39.7 (35.1–44.5)	22.8 (18.4–27.6)	78.0 (71.9–83.3)
German diabetes personal score	0.595†	64.5 (56.4–72.0)	50.9 (48.1–53.7)	14.2 (11.7–17.1)	91.9 (89.6–93.8)	0.587†	81.4 (73.5–87.9)	27.3 (23.2–31.8)	24.5 (20.4–29.0)	83.6 (76.3–89.3)
PREDIMED-clinical Score	0.784	72.2 (64.5–79.1)	72.5 (70.0–75.1)	25.0 (21.1–29.3)	95.4 (93.8–96.6)	0.662	85.4 (78.0–91.2)	26.6 (22.5–31.1)	25.2 (21.2–29.7)	86.4 (79.3–91.7)
FINDRISC clinical score	0.716†	73.5 (65.9–80.3)	62.07 (59.3–64.8)	19.7 (16.5–23.2)	94.9 (93.1–96.3)	0.612†	78.2 (69.9–85.1)	27.3 (23.2–31.8)	23.8 (19.7–28.2)	81.2 (73.9–87.3)

*Abbreviations: AUC, area under the receiver operating characteristic curve; CI, confidence interval; PPV, positive predictive value. The scores were calculated based on different variables included in each of them. Clinical scores include fasting plasma glucose (mg/L) as a variable.*

* *P value<0.05 compared to PREDIMED-personal score,*

† *P value<0.05 compared to PREDIMED-clinical score.*

When we analyzed the ROC curves of several scores in the validation sample (DE-PLAN-CAT), we observed that our clinical score also had a higher AUC than other models (AUC = 0.66). In comparison to the FINDRISC (AUC = 0.61) and the GDRS (AUC = 0.58) scores, our score had a higher predictive ability (P<0.001) in this external validation sample. As expected, the AUC for our personal models were lower when tested in the validation sample (DE-PLAN-CAT) than in the derivation sample (PREDIMED cohort; for our personal score, the AUC was of 0.50, and for the FINDRISC personal score it was 0.52). When only individuals at the same range of age and BMI as in the PREDIMED study were considered as validation sample, the AUC of the PREDIMED-clinical Score remain significantly higher than those of the FINDRISC (data not shown). There were no statistically significant differences between them. For a cut point of ≥6 of our clinical score, sensitivity was 85.4% and specificity was 26.6% in the cohort in which scores were tested. The positive predictive for this score in the validation sample value was 25.2% and the negative predictive value was 86.4%.

## Discussion

Identifying individuals at high risk of developing T2DM is essential to lead this target population the preventive actions, minimizing human and economic costs of diabetic complications [21]. Therefore, we developed two diabetes risk scores, the PREDIMED-personal Score including only information easily obtained by simple questions; and the PREDIMED-clinical score, which added FPG to the model in order to have a simple and useful tool for predicting the risk of diabetes in clinical practice. The present study shows that the PREDIMED-clinical score had good sensitivity, specificity and negative predictive value but lower positive predictive value for identifying those elderly individuals at high risk of developing diabetes in Spanish population at high cardiovascular risk; and had a higher predictive ability than other common diabetes risk scores used in clinical settings.

As far as we know, our study is the first to have analyzed classical predictors of diabetes in an elderly Mediterranean population at high cardiovascular risk. This is important because previous diabetes risk scores were developed in healthy European populations in which lifestyle and ethnicities, the main factors that influence the risk of developing diabetes, differ from those of elderly Spanish individuals, thus limiting their applicability [9], [12], [22].

The diabetes risk scores derived from this subset of the PREDIMED cohort are simple to apply in clinical practice because they are based on variables routinely gathered in primary care, and FPG. They avoid, therefore, the complex and time-consuming biochemical measurements used in other clinical scores [13]. FPG was included in our clinical score because it can considerably improve the performance of models based on non-invasive measures [23]. Thus, it gives clinicians the opportunity to choose the score depending on the availability of FPG.

As in other published studies, BMI, family history of T2DM and hypertension have been identified as risk factors of T2DM. Other lifestyle-related factors that have been established as increasing the risk of diabetes [8], [9], [11], [12], [24], such as smoking or alcohol consumption, have also been identified as significant predictors of incident diabetes in our study.

Although age has been considered as a major risk factor for T2DM in several studies, including the San Antonio Heart Study [12], the GDRS [11] and the FINDRISC analysis [8], it was not significantly associated with a higher risk of incident diabetes in our population. This might be because the age range in our study was, by design, too narrow. Nevertheless, our results in relation to age are consistent with other studies which showed a non-significant association within these variables in the results of their multivariate regression models [23].

The FINDRISC diabetes score, developed in a random Finnish population sample of 35 to 64 year-old men and women, included risk factors that were similar to the PREDIMED diabetes risk scores (BMI, antihypertensive medication, history of blood glucose) [8]. For this reason, and because it is widely used for the screening of individuals at high risk of T2DM, we tried to assess its performance in our population. The GDRS, which was used in the EPIC-Potsdam study in a population of individuals aged 55–65 years, also included risk factors which had been considered in PREDIMED scores, such as alcohol consumption, hypertension or smoking [11]. In the present study, the AUC for the PREDIMED-clinical score was 0.78; in the ARIC study, the AUC for the clinical score, which included variables similar to those in our model (parental history of diabetes, blood pressure and FPG) was also 0.78 [9]. In the PREDIMED-clinical score, a cut-off point of 6 gave a sensitivity of 72.2% and a specificity of 72.5%, and so was optimal for identifying individuals at risk of developing type 2 diabetes in an elderly population at high cardiovascular risk.

When we tried to test PREDIMED diabetes risk scores in an independent external validation sample, the AUC was slightly lower than the estimates obtained in the population in which the score was derived. However, validating risk scores in different populations is likely to result in poorer performance, so the present study is consistent with the results of previous studies [13]. This was observed when the FINDRISC was tested in The Netherlands [25] and when the Framingham offspring diabetes risk score [23] was validated in the Botnia Study [26].

One of the strengths of the current study was that our analysis showed that the AUC of the PREDIMED-clinical score was significantly higher in both the derivation and validation sample than that of other scores when applied to the same samples (FINDRISC and GDRS). Moreover, our scores used fewer variables than other scores, thus saving clinicians time and effort.

Another important issue worth mentioning is that in both the studies used for the analyses, the diagnosis of T2DM was confirmed by a second test, thus providing better identification of new cases of T2DM. One of the limitations inherent to other studies is that diabetes was self-reported, which leads to an underestimation of the diagnosis of incident diabetes. We used only three of the earlier cohorts from the PREDIMED trial because they had the longest follow-ups, and the procedures for comprehensively ascertaining new-onset diabetes cases were more complete. In fact, it is reassuring to observe that the incidence of diabetes in our population (11.2%) was similar to that observed in previous studies such as the San Antonio Heart Study (9.2%) [12] and the Diabetes Prevention Program (13%) [27].

Our study has several limitations. First, the analyses were conducted in an elderly population at high risk for CVD, so the results may not be extrapolatable to the general population. Second, the derivation and validation samples differ in general characteristics. However, when we considered as validation sample only subjects of the same range of age and BMI than the derivation population, the performance of our scores did not significantly change. Third, it should be kept in mind that the subjects of our cohorts were allocated to different lifestyle interventions that could partially account for the incidence of new onset diabetes. However in our statistical analyses we adjusted all our estimates for the interventions. In addition, some of the data that the FINDRISC score required to calculate the risk of diabetes was not available in our population, so we had to apply an adapted version of the FINDRISC score. Finally, although our clinical score showed good sensitivity in both the derivation and validation samples, the specificity and PPV was relatively low, as has been observed for other published scores [28], [29].

In summary, in a sample of three PREDIMED centres, we have identified a set of classical diabetes risk factors and developed a diabetes risk score based on anthropometric measurements, lifestyle, and fasting plasma glucose, which is efficient at predicting elderly individuals at high risk of developing T2DM in a population of Spanish Mediterranean individuals at high cardiovascular risk.
